# Vaccines alone are no silver bullets: a modeling study on the impact of efficient contact tracing on COVID-19 infection and transmission in Malaysia

**DOI:** 10.1093/inthealth/ihac005

**Published:** 2022-03-09

**Authors:** Dhesi Baha Raja, Nur Asheila Abdul Taib, Alvin Kuo Jing Teo, Vivek Jason Jayaraj, Choo-Yee Ting

**Affiliations:** Ainqa Health, Lot 7.01 B & C, Menara BRDB, 285 Jalan Maarof, Bukit Bandaraya, 59000 Kuala Lumpur, Malaysia; Ainqa Health, Lot 7.01 B & C, Menara BRDB, 285 Jalan Maarof, Bukit Bandaraya, 59000 Kuala Lumpur, Malaysia; Saw Swee Hock School of Public Health, National University of Singapore, National University Health System, 12 Science Drive 2, #10-01, Singapore 117549; Department of Social and Preventive Medicine, Level 5, Block I, Faculty of Medicine, Universiti Malaya, 50603, Kuala Lumpur, Malaysia; Faculty of Computing and Informatics, Multimedia University, Persiaran Multimedia, 63100 Cyberjaya, Selangor, Malaysia

**Keywords:** contact tracing, COVID-19, transmission, vaccination

## Abstract

**Background:**

The computer simulation presented in this study aimed to investigate the effect of contact tracing on coronavirus disease 2019 (COVID-19) transmission and infection in the context of rising vaccination rates.

**Methods:**

This study proposed a deterministic, compartmental model with contact tracing and vaccination components. We defined contact tracing effectiveness as the proportion of contacts of a positive case that was successfully traced and the vaccination rate as the proportion of daily doses administered per population in Malaysia. Sensitivity analyses on the untraced and infectious populations were conducted.

**Results:**

At a vaccination rate of 1.4%, contact tracing with an effectiveness of 70% could delay the peak of untraced asymptomatic cases by 17 d and reduce it by 70% compared with 30% contact tracing effectiveness. A similar trend was observed for symptomatic cases when a similar experiment setting was used. We also performed sensitivity analyses by using different combinations of contact tracing effectiveness and vaccination rates. In all scenarios, the effect of contact tracing on COVID-19 incidence persisted for both asymptomatic and symptomatic cases.

**Conclusions:**

While vaccines are progressively rolled out, efficient contact tracing must be rapidly implemented concurrently to reach, find, test, isolate and support the affected populations to bring COVID-19 under control.

## Introduction

The pandemic caused by severe acute respiratory syndrome coronavirus 2 (SARS-CoV-2) has infected >200 million people and led to 4.5 million deaths worldwide, as of 26 August 2021.^1^ Since January 2021, approximately 2 billion people have been fully vaccinated against coronavirus disease 2019 (COVID-19), with several countries, mostly upper middle-income economies, having reached 70% full vaccination to date.^1,2^ Despite this, the pandemic shows no sign of abatement, with the spread of the delta variant triggering new outbreaks in many countries globally.^3^

Governments of various countries have started to invest in digital contact tracing since 2020. Digital contact tracing is an approach to disrupt the chains of disease transmission by identifying and isolating those in close contact with an infected individual.^4^ It leverages proximity and geospatial technologies to provide a comprehensive approach to collect spatio-temporal data.^5,6^ The data can be used to study the movement and interaction of humans across time, which can then be utilized further for investigating disease transmissions such as COVID-19, TB and other communicable diseases. Successful digital contact tracing can keep the reproduction number (${R_0}$) under control, while failing to have efficient digital contact tracing could cause the number of daily cases to increase exponentially. A study conducted by Mizumoto et al. has shown that, for COVID-19, 70% of transmissions occur before someone is symptomatic,^7^ indicating the importance of having a speedy and accurate contact tracing mechanism. Another study performed by Abueg et al. has also reported that if digital contact tracing is used by 75% of the population, the number of infections can be reduced by 73–79%.^8^

Among those fully vaccinated, reports of breakthrough infections, severe illness and deaths have since been reported in countries like Iran and Indonesia.^9,10^ The evidence on the protective effect of several COVID-19 vaccines in the WHO Emergency Use Listing against the delta variant has gradually emerged, with their reported effectiveness being lower than the protection conferred against the alpha variant.^11–13^ Nevertheless, the evidence thus far indicates that vaccines are effective against symptomatic and severe COVID-19,^12,14,15^ and vaccine uptake and administration should be ramped up globally. From a public health perspective, it is vital to reduce the transmission and incidence of infection to protect pockets of populations that could not be vaccinated and to allow economies to open in a safe and calibrated manner.

As of 26 August 2021, the cumulative number of COVID-19 cases in Malaysia exceeded 1.5 million, and the daily new confirmed cases per 100 000 population remains one of the highest in the world.^1^ Although Malaysia has consistently rolled out 400 000–500 000 doses of vaccines per day since July 2021 and implemented multiple iterations of movement control orders with different measures since March 2020, the pandemic continues to rage, with a record number of cases and deaths daily. Contact tracing efforts are also severely hampered due to the strain on the public health system, resulting in missed contacts, who might have been infected but did not know their risk, to delay testing and further transmit the virus.^16,17^

It is increasingly evident that a single intervention, be it vaccine, public health or social measures, is insufficient to control COVID-19. Hence, in this theoretical strategy exploration study, we aimed to investigate the importance of implementing effective contact tracing on COVID-19 transmission and infection in the context of rising vaccination rates using a deterministic, compartmental modeling approach as an experimental basis for our discussion.

## Materials and Methods

### Design

First, we proposed a novel transmission model that factored in contact tracing effectiveness and vaccination. Next, we determined the parameters using estimations based on Malaysian COVID-19 data and information from published literature. Finally, we conducted sensitivity analyses of the parameters on the number of untraced, infectious individuals.

### Epidemic model

We developed a deterministic, compartmental susceptible-exposed-infected-recovered-vaccinated (SEIRV) model to study the transmission dynamics of COVID-19 when contact tracing and vaccination were incorporated. The human population was subdivided into 10 classes according to their disease status, namely, the susceptibles (*S*), exposed (*E*), traced exposed (*T*), quarantined symptomatic infected (${Q^{sym}}$), quarantined asymptomatic infected (${Q^{asym}}$), symptomatic infected (${I^{sym}}$), asymptomatic infected (${I^{asym}}$), recovered (*R*), death (*D*) and vaccinated (*V*). The susceptible compartment (*S*) was composed of all healthy individuals who could get infected with SARS-CoV-2. Individuals in the exposed compartment (*E*) were those who had been infected with the virus but remained in their latent period and untraced. The traced exposed (*T*) compartment referred to infectives in their latent period who were successfully traced and isolated. The quarantined symptomatic infected (${Q^{sym}}$) compartment comprised infected individuals who were infectious with symptoms and in quarantine either at home or in hospital, whereas the quarantined asymptomatic infected (${Q^{asym}}$) compartment was composed of those infectious individuals without symptoms and in quarantine. Individuals in the symptomatic infected (${I^{sym}}$) compartment referred to those untraced infectious individuals who have developed the symptoms, while those untraced infectious individuals without symptoms belonged in the asymptomatic infected (${I^{asym}}$) compartment. Those who recovered with COVID-19 immunity made up the recovered (*R*) compartment, and those who received COVID-19 immunity through vaccination formed the vaccinated (*V*) compartment. Lastly, victims who died from COVID-19 were represented by the compartment *D*. Individuals could transition from one compartment over time but were only allowed to be in one compartment at a time. These compartments are summarized in Table 1.

**Table 1.** Description of compartments in the SEIRV model

State variable	Description
*S*	Susceptible individuals
*T*	Infected individuals in latent period (exposed) that were traced and quarantined
*E*	Infected individuals in latent period (exposed) that were untraced
${Q^{sym}}$	Symptomatic infectious individuals that were traced and quarantined
${Q^{asym}}$	Asymptomatic infectious individuals that were traced and quarantined
${I^{sym}}$	Symptomatic infectious individuals that were untraced
${I^{asym}}$	Asymptomatic infectious individuals that were untraced
*R*	Recovered individuals with immunity
*D*	Deaths due to COVID-19
*V*	Vaccinated individuals

### Model assumptions

The susceptibles (*S*) could become infectives when they met with either symptomatic (${I^{sym}}$) or asymptomatic (${I^{asym}}$) infectious individuals at different transmission rates of ${\beta _{sym}}$ or ${\beta _{asym}}$, respectively. This transmission rate was the product of contact rate and the probability of transmission given contact. In this paper, we assumed that these public health and social measures (PHSM) did not vary across time and that the population was not partitioned according to age or comorbidity. Also, natural births and deaths were not considered. Our focus was on analyzing the ‘trace and isolate’ policy, whereby tracing could be done manually or through an automated process using tracing apps. Hence, we subdivided the exposed compartment (infectives in the latent period) further into traced exposed (*T*) and untraced exposed (*E*) compartments, that is, compartments $( {E,\ T} )$ are the subpopulations of the exposed population separated according to their contact tracing status. The implementation of the ‘trace and isolate’ approach could reduce the transmission of COVID-19 by forcing the traced exposed individuals into quarantine (*T*) through self-isolation. Therefore, we assumed that all the traced individuals in quarantine (*T*) will have full compliance.

As they were unaware of their disease status, asymptomatic infectious individuals (${I^{asym}}$) would continue to contribute to the transmission of the virus when they met another susceptible at a rate of ${\beta _{asym}}$, leading to untraced infectives in the *E* compartment. Following the work of Grimm and colleagues,^18^ the parameter $\omega $ denoted the proportion of the symptomatic infectious population (${I^{sym}}$) detected by the health authorities out of all symptomatic infections, while the parameter $\tau $ was the contact tracing effectiveness that described the fraction of the contacts traced either manually or via digital tools such as tracing apps. Hence, the product $\omega \tau $ gave the total proportion of infectives in their latent period who were successfully traced and transitioned into the (*T*) compartment at a rate of ${\beta _{sym}}$, whereas (1–$\omega \tau $) referred to tracing failures and, thus, (1–$\omega \tau $) proportion of infectives would enter the untraced exposed (*E*) compartment at a rate of ${\beta _{sym}}$.

**Figure 1.**
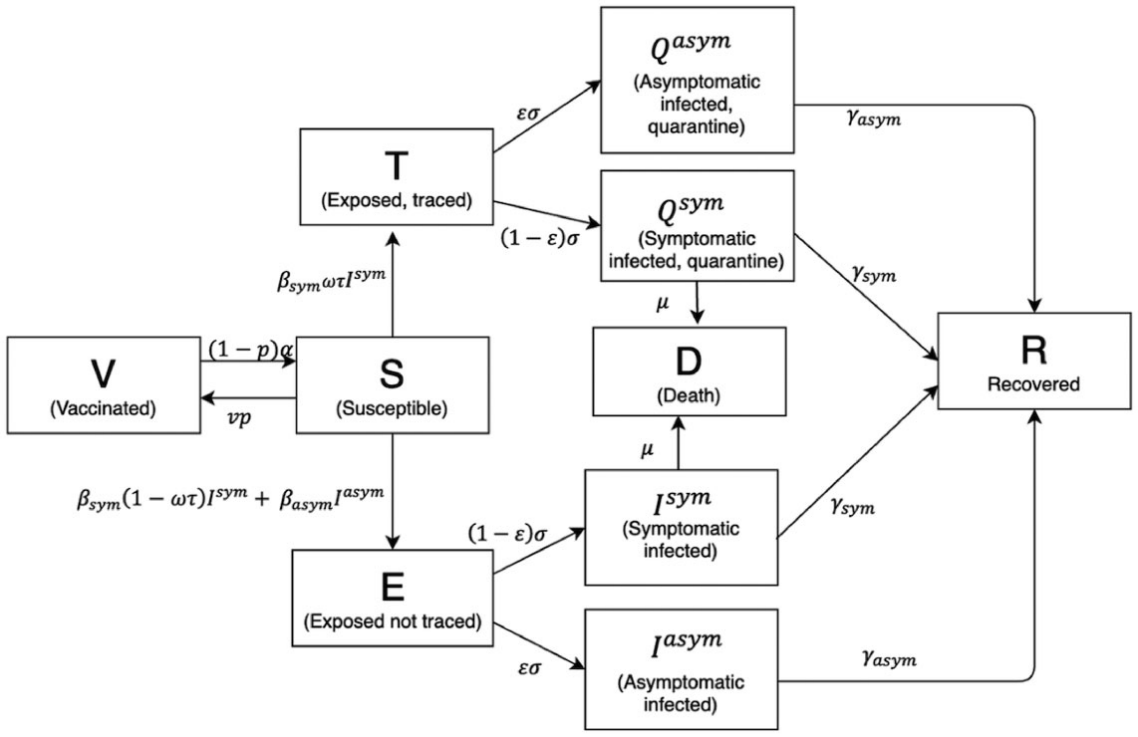
Transmission diagram of the SEIRV model.

Furthermore, infectives in their latent period ($E,\ T$) would become infectious at a rate of $\sigma $, which denoted the reciprocal of the latent period. With $\varepsilon $ as the fraction of exposed individuals ($E,\ T$) who were asymptomatic, then $\varepsilon \sigma E$ would be the number of untraced exposed (*E*) entering the asymptomatic infectious compartment (${I^{asym}}$), while $\varepsilon \sigma T$ referred to the number of traced exposed (*T*) who would move into the asymptomatic infectious quarantine (${Q^{asym}}$) compartment. This gives (1–$\varepsilon $) as the fraction of the exposed population ($E,\ T$) who were symptomatic, which leads to (1–$\varepsilon )\sigma E$, the number of untraced exposed (*E*) moving into the symptomatic infectious compartment (${I^{sym}}$), whereas (1–$\varepsilon )\sigma T$ is the number of traced exposed (*T*) entering the asymptomatic infectious quarantine (${Q^{sym}}$) compartment. We assumed that only symptomatic infected individuals could die from COVID-19 at a rate of $\mu $ thus entering the *D* compartment. We also assumed that the disease-induced death rate ($\mu $) was constant and unaffected by disease severity and hospital capacity. Symptomatic infected individuals (${I^{sym}},\ {Q^{sym}}$) would recover at a rate of ${\gamma _{sym}}$, which was the reciprocal of the duration of infectiousness of symptomatic patients. On the other hand, asymptomatic infected individuals (${I^{asym}},\ {Q^{asym}}$) would recover at a rate of ${\gamma _{asym}}$, which was the reciprocal of the duration of infectiousness of asymptomatic patients. Finally, we incorporated vaccination into the model. In this work, the vaccine functioned by reducing the number of susceptibles, thus preventing further infections at a rate of $vp$, which referred to the product of vaccination coverage and the probability of vaccination success. The parameters (1–*p*) and $\alpha $ denoted the probability of vaccination failure in preventing transmissions and the reciprocal duration for the waning of vaccine immunity, respectively. Hence, (1–$p)\alpha V$ gave the number of vaccinated individuals who would move back to being susceptibles (*S*). We assumed that the vaccinated individuals gain perfect protection and will not be infected after interacting with an infectious individual, except for when they have lost their acquired immunity due to the probability of vaccination failure (1–$p)$. The dynamics of transmission are visualized in Figure 1, with the description of parameters listed in Table 2.

**Table 2.** Parameter descriptions

Parameter	Description
${\beta _{sym}}$	Transmission rate of symptomatic individuals per day per case
${\beta _{asym}}$	Transmission rate of asymptomatic individuals per day per case
$\omega $	Fraction of symptomatic individuals identified by health authorities per day
$\tau $	Fraction of contacts traced per case either manually or via digital tools
$\sigma $	Reciprocal of the latent period
${\gamma _{asym}}$	Reciprocal of the infectious period of asymptomatic individuals
${\gamma _{sym}}$	Reciprocal of the infectious period of symptomatic individuals
$\varepsilon $	Fraction of asymptomatic infectious individuals per day
$\mu $	Disease-induced death rate
*v*	Vaccination coverage per day
*p*	Probability of vaccination success
$\alpha $	Reciprocal of the time taken for waning vaccine immunity

### Model equations

Following the previous assumptions, our SEIRV model was described by a non-linear system of 10 ordinary differential equations with 12 parameters:

$$\begin{eqnarray*}
\frac{{dS}}{{dt}} &=& \left( {1 - p} \right)\ \alpha V - ( {{\beta _{sym}}\omega \tau S{I^{sym}} + \ {\beta _{asym}}S{I^{asym}}}\nonumber\\
&&+\, {\beta _{sym}}\left( {1 - \omega \tau } \right)S{I^{sym}} + vpS ),
\end{eqnarray*}$$

$$\begin{equation*}{\rm{\ }}\frac{{dT}}{{dt}} = {\beta _{sym}}\ \omega \tau S{I^{sym}} - \sigma T,\end{equation*}$$

$$\begin{equation*}{\rm{\ }}\frac{{dE}}{{dt}} = \ {\beta _{sym}}\left( {1 - \omega \tau } \right)S{I^{sym}} + \ {\beta _{asym}}S{I^{asym}} - \sigma E,\end{equation*}$$

$$\begin{equation*}{\rm{\ }}\frac{{d{Q^{sym}}}}{{dt}} = \left( {1 - \varepsilon } \right)\ \sigma T - \left( {{\gamma _{sym}} + \mu } \right){Q^{sym}},\end{equation*}$$

$$\begin{equation*}{\rm{\ }}\frac{{d{Q^{asym}}}}{{dt}} = \ \varepsilon \sigma T - {\gamma _{asym}}{Q^{asym}},\end{equation*}$$

$$\begin{equation*}{\rm{\ }}\frac{{d{I^{sym}}}}{{dt}} = \left( {1 - \varepsilon } \right)\ \sigma E - \left( {{\gamma _{sym}} + \mu } \right){I^{sym}},\end{equation*}$$

$$\begin{equation*}{\rm{\ }}\frac{{d{I^{asym}}}}{{dt}} = \ \varepsilon \sigma E - {\gamma _{asym}}{I^{asym}},\end{equation*}$$

$$\begin{equation*}{\rm{\ }}\frac{{dR}}{{dt}} = {\gamma _{sym}}\ \left( {{Q^{sym}} + {I^{sym}}} \right) + {\gamma _{asym}}\left( {{Q^{asym}} + {I^{asym}}} \right),\end{equation*}$$

$$\begin{equation*}{\rm{\ }}\frac{{dD}}{{dt}} = \ \mu \left( {{I^{sym}} + {I^{asym}}} \right)\ {\rm{and}}\end{equation*}$$

$$\begin{equation*}{\rm{\ }}\frac{{dV}}{{dt}} = \ vpS - \left( {1 - p} \right)\alpha V,\end{equation*}$$

with initial conditions $S{\rm{\ }}( 0 ) = {\rm{\ }}{S_0} > 0,{\rm{\ }}T > 0,{\rm{\ }}E{\rm{\ }}( 0 ) = {\rm{\ }}{E_0} > 0,$${Q^{sym}}{\rm{\ }}( 0 ) = {Q^{sym}}{{\rm{\ }}_0} > 0,{\rm{\ }}{Q^{asym}}{\rm{\ }}( 0 ) = {Q^{asym}}{{\rm{\ }}_0} > 0,{\rm{\ }}{I^{sym}}{\rm{\ }}( 0 ) = {I^{sym}}{{\rm{\ }}_0} > 0,$${I^{asym}}{\rm{\ }}( 0 ) = {I^{asym}}{{\rm{\ }}_0} > 0,$$R{\rm{\ }}( 0 ) = {\rm{\ }}{R_0} > 0,$$D{\rm{\ }}( 0 ) = {\rm{\ }}{D_0} > 0,$ and $V{\rm{\ }}( 0 ) = {\rm{\ }}{V_0} > 0$.

### Parameter estimations

The values of parameters in this paper were either estimated using Malaysian COVID-19 data or adapted from the literature. From a COVID-19 modeling study in Malaysia conducted by Gill et al.,^19^ we took the average contacts per day per case (n=25) and the probability of transmission given contact with symptomatic individuals (=0.05), whereby the transmission probability was based on the infectiousness of the original COVID-19 strain during early 2020. We calculated the transmission rate $\beta $ as the product of the two, which gave us 25×0.05=1.25. However, because we assumed that the population was closed with a constant size (N=32 600 000; i.e., the total Malaysian population), we divided the transmission rate $\beta \ $by N to get our final ${\beta _{sym}}$ as $3.8 \times {10^{ - 8}}$. To calculate ${\beta _{asym}},\ $we followed the same steps but replaced the probability of transmission given contact with asymptomatic individuals (=0.02) from Churches and Jorm,^20^ to get the ${\beta _{asym}}$ as $1.5 \times {10^{ - 8}}.$

We defined the effectiveness of contact tracing as the proportion of contacts of a positive case that was successfully traced. In Malaysia, we estimated that the proportion of contacts of a COVID-19 case traced per case varied from 30 to 40%.^21,22^ Hence, we have taken the midpoint of the interval (35%) as the estimated contact tracing effectiveness in Malaysia in one of our simulation scenarios (i.e., scenario 4). The vaccination coverage per day (*v*) was estimated from Malaysian vaccination statistics, where around 1.0–1.5% of the total population were vaccinated daily. Also, we included the values for vaccination coverage (*v*=0.8%) and contact tracing effectiveness ($\tau $=50, 70 and 90%), as well as for our simulation exercise. Other parameters obtained from the literature review are summarized in Table 3.

**Table 3.** Parameter values

Parameter	Value	Source
${\beta _{sym}}$	$3.8 \times {10^{ - 8}}$	^19^
${\beta _{asym}}$	$1.5 \times {10^{ - 8}}$	^20^
$\omega $	0.9	^19^
$\tau $	0.9, 0.7, 0.5, 0.4, 0.3	estimated, dynamic
$\sigma $	1/5	^18^
${\gamma _{asym}}$	1/10	^18^
${\gamma _{sym}}$	1/12.5	^18^
$\varepsilon $	0.25	^18^
$\mu $	0.02	^20^
*v*	0.014, 0.012, 0.01, 0.008	estimated, dynamic
*p*	0.9	^29^
$\alpha $	1/30	^29^

### Sensitivity analysis

In order to solve our SEIRV model, we implemented a numerical integration method, Runge–Kutta of order 5, by using the *solve_ivp* function from the *scipy.integrate* module in Python (Python Software Foundation, version 2.7) along with the parameter values in Table 3. Next, to study the dynamics of transmission of COVID-19 concerning contact tracing and vaccination, we conducted sensitivity analyses of the parameters on the untraced, infectious individuals. We were interested in observing the simulation on ${I^{asym}}\ $and ${I^{sym}}$ as these populations would contribute to forward transmission because they were not traced and isolated. Hence, an uncontrolled number of ${I^{asym}}\ $and ${I^{sym}}$ would in turn lead to a potential surge in future total COVID-19 cases. We varied the values of vaccination coverage *v* and contact tracing effectiveness $\tau $ and investigated the effects of the changes on ${I^{asym}}\ $and ${I^{sym}}$. We prepared four scenarios, as summarized in Table 4.

**Table 4.** Scenarios for sensitivity analysis

Scenario	Fixed parameter	Value (/day)	Varying parameter(s)	Value (/day)
1	Vaccination coverage	1.4%	Contact tracing effectiveness	30%, 40%, 50%, 70%, 90%
2	—		(Contact tracing effectiveness, vaccination coverage)	(30%, 1.5%), (40%, 1.4%), (50%, 1.3%), (70%, 1.2%), (90%, 1%)
3	Contact tracing effectiveness	90%	Vaccination coverage	0.8%, 1%, 1.3%, 1.4%
4	Contact tracing effectiveness	35%	Vaccination coverage	0.8%, 1%, 1.3%, 1.4%

## Results

**Figure 2.**
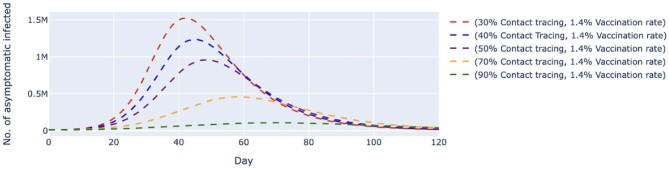
Simulated number of untraced, COVID-19 asymptomatic cases with fixed vaccination rate and varied contact tracing effectiveness. The vaccination rate per day was fixed at 1.4%, which translates to approximately 450 000 doses of vaccine, and the contact tracing effectiveness varied from 30 to 90%. The dashed lines represent the simulated number of untraced COVID-19 asymptomatic cases for five different contexts over 120 d. M, million.

**Figure 3.**
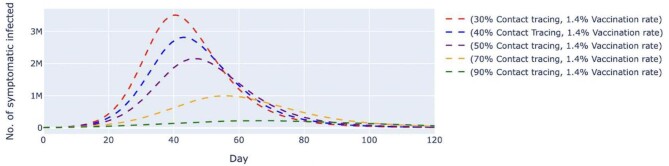
Simulated number of untraced, COVID-19 symptomatic cases with fixed vaccination rate and varied contact tracing effectiveness. The vaccination rate per day was fixed at 1.4%, which translates to approximately 450 000 doses of vaccine, and the contact tracing effectiveness varied from 30 to 90%. The dashed lines represent the simulated number of untraced COVID-19 symptomatic cases for five different contexts over 120 d. M, million.

Scenario 1 simulated five different contact tracing effectiveness values ranging from 30 to 90% against the backdrop of a fixed vaccination rate of 1.4% per day (Figures 2 and 3). This scenario assumed that the vaccine was administered at a rate of approximately 450 000 doses per day. We found that when contact tracing effectiveness was at 30%, the number of untraced, asymptomatic cases would peak at day 42 with approximately 1.52 million cases before gradually slowing down; the peak decreases as the effectiveness of contact tracing increases (Figure 2). When contact tracing effectiveness was increased to 70%, the peak was delayed by about 17 d, with the highest number of daily cases at 459 000, which was about a 70% reduction from the number estimated when contact tracing effectiveness was at 30%. It can be observed that a contact tracing effectiveness of 90% would almost flatten the curve. Similar behavior could be observed for the untraced, symptomatic cases in Figure 3, in which a combination of a high vaccination rate (1.4%) and low contact tracing effectiveness (30%) would cause the peak of the cases to be at about 3.5 million on day 40. When we increased the contact tracing effectiveness to 70%, the peak was delayed by about 15 d, with approximately 1 million cases, which was about 29% of those estimated when contact tracing was at 30% effectiveness. When contact tracing effectiveness increased, the peaks for both cases were delayed and lowered.

**Figure 4.**
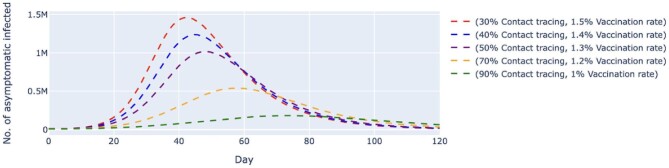
Simulated number of untraced, COVID-19 asymptomatic cases with varied vaccination rates and contact tracing effectiveness. The vaccination rates per day varied from 1 to 1.5%, which translates to approximately 320 000 and 480 000 doses of vaccine, respectively. The contact tracing effectiveness varied from 30 to 90%. The dashed lines represent the simulated number of untraced COVID-19 asymptomatic cases for five different contexts over 120 d. M, million.

**Figure 5.**
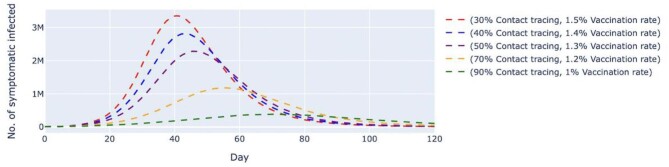
Simulated number of untraced, COVID-19 symptomatic cases with varied vaccination rates and contact tracing effectiveness. The vaccination rates per day varied from 1 to 1.5%, which translates to approximately 320 000 and 480 000 doses of vaccine, respectively. The contact tracing effectiveness varied from 30 to 90%. The dashed lines represent the simulated number of untraced COVID-19 symptomatic cases for five different contexts over 120 d. M, million.

In scenario 2, we conducted simulations by pairing higher contact tracing effectiveness with a lower daily vaccination rate and vice versa. Despite a higher vaccination rate, the simulated trend of new daily untraced, asymptomatic cases contingent on 30% contact tracing effectiveness was estimated to peak at day 42 with 1.46 million cases. The variant with 90% contact tracing effectiveness but a lower vaccination rate delayed peaking at day 74 with 182 000 cases (Figure 4). The same trends were observed among symptomatic cases (Figure 5). A low contact tracing effectiveness of 30%, with a high daily vaccination rate of 1.5%, would lead to a peak of 3.35 million untraced, symptomatic cases on day 41. The scenario with the combination of 90% contact tracing effectiveness and a vaccination rate of only 1% managed to delay the peak to day 72 with 381 000 cases.

**Figure 6.**
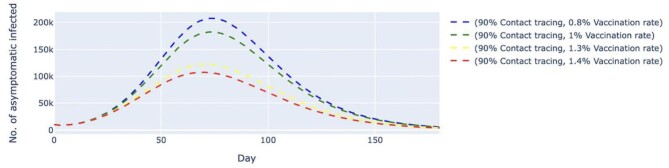
Simulated number of untraced, COVID-19 asymptomatic cases with fixed contact tracing effectiveness at 90% and varied vaccination rates. The contact tracing effectiveness was fixed at 90% and the vaccination rates per day varied from 0.8 to 1.4%, which translates to approximately 260 000 and 450 000 doses of vaccine, respectively. The dashed lines represent the simulated number of untraced COVID-19 asymptomatic cases for five different contexts over 160 d. k, thousand.

**Figure 7.**
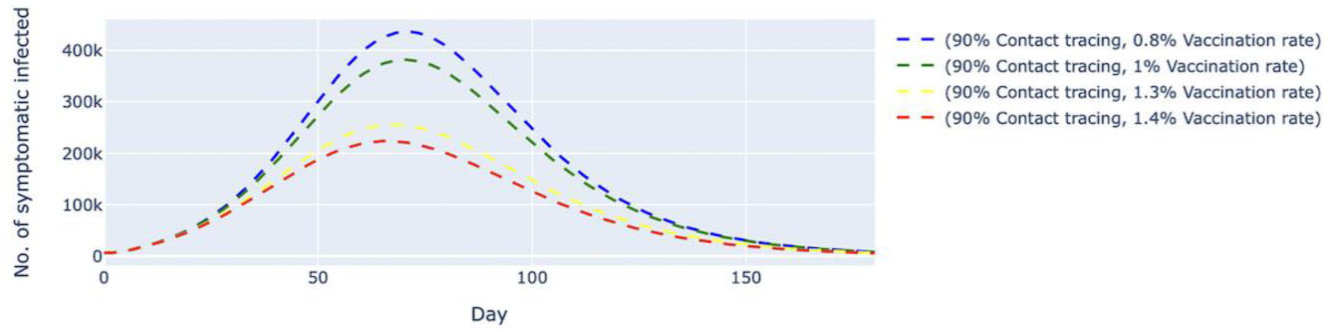
Simulated number of untraced, COVID-19 symptomatic cases with fixed contact tracing effectiveness at 90% and varied vaccination rates. The contact tracing effectiveness was fixed at 90% and the vaccination rates per day varied from 0.8 to 1.4%, which translates to approximately 260 000 and 450 000 doses of vaccine, respectively. The dashed lines represent the simulated number of untraced COVID-19 symptomatic cases for five different contexts over 160 d. k, thousand.

**Figure 8.**
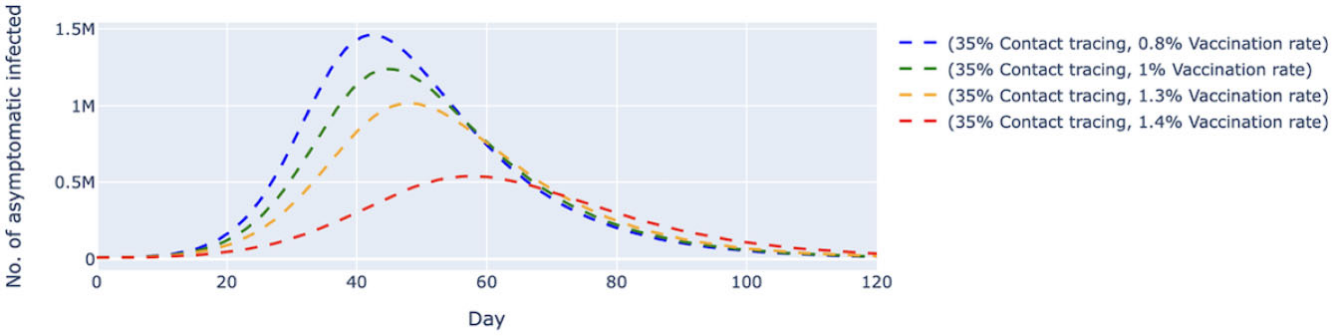
Simulated number of untraced, COVID-19 asymptomatic cases with fixed contact tracing effectiveness at 35% and varied vaccination rates. The contact tracing effectiveness was fixed at 35%, and the vaccination rates per day varied from 0.8 to 1.4%, which translates to approximately 260 000 and 450 000 doses of vaccine, respectively. The dashed lines represent the simulated number of untraced COVID-19 asymptomatic cases for five different contexts over 120 d. M, million.

**Figure 9.**
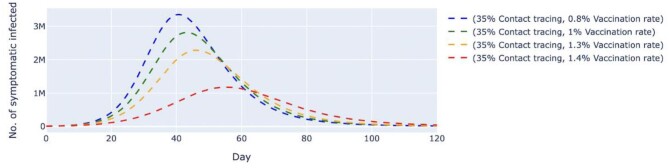
Simulated number of untraced, COVID-19 symptomatic cases with fixed contact tracing effectiveness at 35% and varied vaccination rates. The contact tracing effectiveness was fixed at 35%, and the vaccination rates per day varied from 0.8% to 1.4%, which translates to approximately 260 000 and 450 000 doses of vaccine, respectively. The dashed lines represent the simulated number of untraced COVID-19 symptomatic cases for five different contexts over 120 d. M, million.

We also simulated two other scenarios where the contact tracing effectiveness was fixed at 90% (Figures 6 and 7) and 35% (Figures 8 and 9), respectively. In both scenarios, the vaccination rates varied from 0.8 to 1.4% per day. At 90% contact tracing effectiveness (Figure 6), the peaks for all permutations occurred at around the same time (day 68 to day 73) and ranged from 107 000 to 207 000 cases. The estimated highest number of daily untraced, asymptomatic cases differed by approximately 100 000 between high and low vaccination rates when contact tracing effectiveness was fixed. The increase in the vaccination rate could only delay the peak but failed to lower the peak significantly. For the untraced symptomatic cases in Figure 7, a combination of a low vaccination rate (1%) and high contact tracing effectiveness (90%) could successfully reduce the highest number of daily cases to about 381 000 at day 70. Under the same circumstances, except for a lower contact tracing effectiveness (Figure 8), the estimated number of daily untraced asymptomatic cases was 540 000 on day 57 when the vaccination rate was 1.4%. A similar trend could be seen for the untraced, symptomatic cases in Figure 9, whereby a high vaccination rate (1.4%) but low contact tracing effectiveness (35%) could still cause the peak of cases to be 1.2 million on day 54. Furthermore, results from an additional sensitivity analysis on the transmission rate of symptomatic infected cases can be found in the supplementary material. The simulation showed that the number of daily cases was highly affected by the transmission rates of symptomatic infected, even when the daily vaccination rate and contact tracing effectiveness were high. Lower transmission rates, which indicate effective social distancing measures, would reduce and delay the peak of daily cases.

## Discussion

The pandemic saw major movement and travel restrictions, lockdowns and personal protective measures, implemented globally in various forms and stringency. The PHSM are effective in limiting COVID-19 transmission and death.^23^ However, some of the interventions, particularly lockdowns and a cessation of economic activities, have negatively impacted the economies and psychosocial well-being of the affected populations.^24,25^ One major component of PHSM is the enhancement of surveillance and response actions through contact tracing, testing and isolating close contacts, as well as providing the necessary support mechanisms.^23^

In this paper, we simulated the impact of contact tracing on COVID-19 transmission and infection in the context of rising vaccination rates. We observed that a combination strategy of a high daily vaccination rate and low contact tracing effectiveness would significantly increase the untraced infectious population. While the vaccine has been presented as one of the most vital tools to take us towards the restoration of postpandemic normality, we found that contact tracing is key to COVID-19 control. However, the ability of countries to perform contact tracing is challenged by a lack of human resources (contact tracers) and compliance with self-isolation orders, as well as a paucity of timely and accurate contacts data. Successes observed in countries like Singapore and South Korea leveraged technology to aid contact tracing,^6^ and the integration of digital technology with the conventional contact tracing approach could result in a swifter response to stem COVID-19 transmission.

In Malaysia, personal details are collected for entry to all premises outside of one's residence using a mobile application (MySejahtera) or are documented in writing for contact tracing purposes.^26,27^ However, the contact tracing of private social gatherings in homes remains an issue and has to be carried out manually, therefore hampering the effectiveness of contact tracing. With the rapid increase in COVID-19 infections and an overwhelmed public health system, the effectiveness of contact tracing in Malaysia has been hindered and currently stands at approximately 30 to 40%.^21,22^ Given the significance of contact tracing in controlling the spread of infectious diseases, it is pivotal to capitalize contacts data and automate the data processing and application process,^4^ as well as to develop more efficient strategies to improve the effectiveness of contact tracing.

In this study, we modeled the number of untraced cases, both symptomatic and asymptomatic. Because this population contributes to forward transmission, an uncontrolled number of these untraced and unisolated individuals could potentially cause a surge in COVID-19 cases. In addition to transmission and infection, future work could be considered using an extended model that incorporates disease severities and health system capacity to estimate the effect of contact tracing on COVID-19 mortality. Furthermore, we set the two main intervention parameters—contact tracing and vaccine—to approximate the ground realities in Malaysia. Notwithstanding testing only on these two PHSMs, we observed that the scenario with low contact tracing and increasing vaccination rates successfully mimicked the current transmission trend in Malaysia. However, it should be noted that this is a theoretical strategy exploration study, as the model was not calibrated to reflect the observed outbreaks in Malaysia and forecast future trends. Hence, further parameterization using local data is warranted to generate outcome estimates more salient to Malaysia.

Contact tracing has been at the forefront in controlling the spread of infectious diseases, and an effective and efficient system could prevent the spread of disease, save lives and allow the economy to resume.^28^ While vaccination rates have progressively increased in Malaysia and some parts of the world, efficient contact tracing must be rapidly implemented to reach, find, test, isolate and support the affected populations to bring COVID-19 under control.

## Conclusions

This study showed that effective contact tracing is a critical component in COVID-19 control. Our findings indicate the need to inform immediate intervention, such as by using technology and automation, in improving the effectiveness and efficiency of the contact tracing strategy to reduce COVID-19 transmission, infection and death in Malaysia and other countries affected by COVID-19.

## Supplementary Material

ihac005_Supplemental_Files

## Data Availability

None.
