# Supplementary figures and images for: Vaccines alone are no silver bullets: a modeling study on the impact of efficient contact tracing on COVID-19 infection and transmission in Malaysia
Source: Int Health. 2022 Mar 9;15(1):37–46. doi: 10.1093/inthealth/ihac005 (PMC8992270; doi:10.1093/inthealth/ihac005)

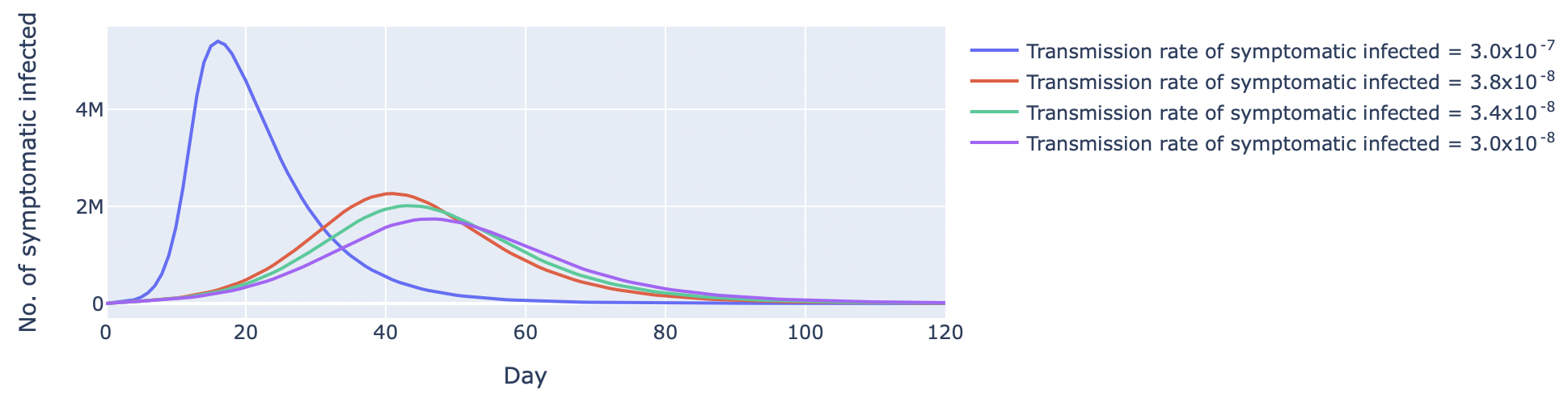

Supplement: ihac005_Supplemental_Files [file ihac005_supplemental_files.zip › Supplementary Figure A1.png]

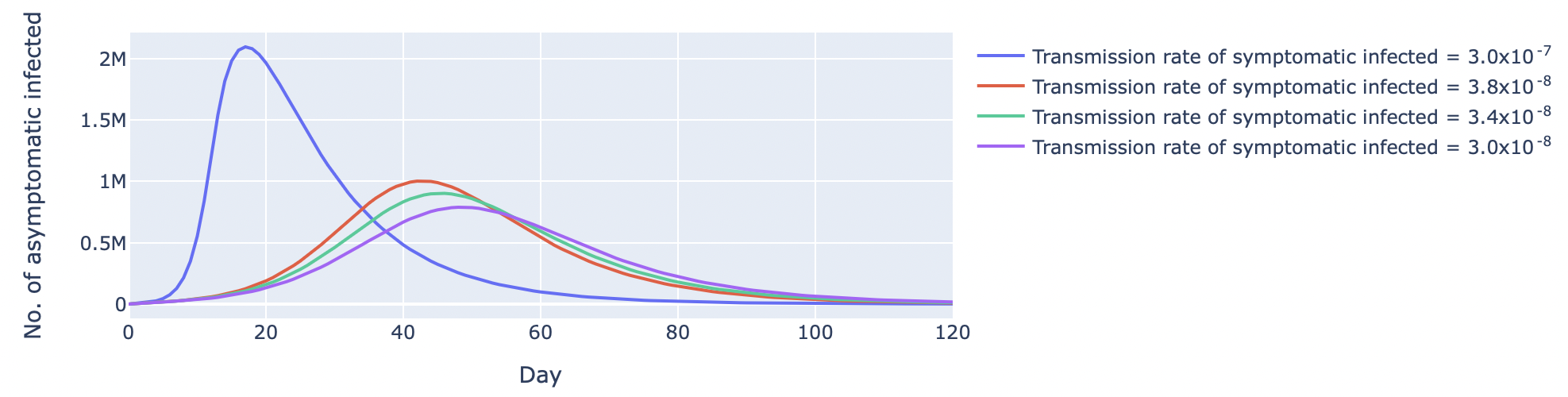

Supplement: ihac005_Supplemental_Files [file ihac005_supplemental_files.zip › Supplementary Figure A2.png]
